# MRI-PDFF Assessment of Intrahepatic Fat Changes Post-Bariatric Surgery: A Systematic Literature Review

**DOI:** 10.3390/medicina60122003

**Published:** 2024-12-04

**Authors:** Danut Dejeu, Paula Dejeu, Anita Muresan, Paula Bradea, Viorel Dejeu

**Affiliations:** 1 Surgical Oncology Department, Emergency County Hospital Oradea, Strada Gheorghe Doja 65, 410169 Oradea, Romania; ddejeu@uoradea.ro (D.D.); amuresan@uoradea.ro (A.M.); 2 Surgery Department, Faculty of Medicine and Pharmacy, University of Oradea, Piata 1 Decembrie 10, 410073 Oradea, Romania; 3 Bariatric Surgery Department, Medlife Humanitas Hospital, Strada Frunzisului 75, 400664 Cluj Napoca, Romania; 4 Laboratory Medicine Unit, Betania Medical Center, Menumorut 12, 410004 Oradea, Romania; 5 Gastroenterology Unit, Betania Medical Center, Menumorut 12, 410004 Oradea, Romania; paula.bradea@betania-centrulmedical.ro; 6 Bariatric Surgery Department, Life Memorial Hospital, Calea Grivitei 365, 010719 Bucuresti, Romania; office@doctordejeu.ro

**Keywords:** bariatric surgery, non-alcoholic fatty liver disease, MRI-PDFF, intrahepatic fat, weight loss

## Abstract

*Background and Objectives*: Non-alcoholic fatty liver disease (NAFLD) is prevalent among obese individuals and can progress to non-alcoholic steatohepatitis (NASH). Bariatric surgery is known to induce significant weight loss and may improve NAFLD. This systematic review uniquely synthesizes current evidence on the effects of bariatric surgery on intrahepatic fat content, measured by magnetic resonance imaging proton density fat fraction (MRI-PDFF), and assesses study quality using the Newcastle–Ottawa Scale (NOS). *Materials and Methods*: The literature search was conducted across the PubMed, Scopus, and Web of Science databases up to October 2024, identifying 12 prospective cohort studies involving 613 patients who underwent bariatric surgery. Inclusion criteria included adult patients with NAFLD undergoing bariatric surgery, assessment of liver fat changes using MRI-PDFF before and after surgery, and studies reporting quantitative data on liver fat fraction and relevant clinical parameters. Data extraction focused on patient demographics, surgical procedures, specific weight loss outcomes (delta BMI), changes in intrahepatic fat content (delta MRI-PDFF), and quality assessment scores based on the NOS. *Results*: Significant reductions in intrahepatic fat content were observed across all studies, with delta MRI-PDFF reductions ranging from 6.9% to 14%. Weight loss outcomes varied, with excess weight loss percentages up to 81.3% and BMI reductions up to 12 kg/m². The quality assessment scores ranged from six to nine out of nine, indicating generally high-quality studies. Correlations were noted between the degree of weight loss and reduction in liver fat content. Several studies reported high rates of resolution of steatosis and NASH post-operatively. *Conclusions*: Bariatric surgery leads to significant reductions in intrahepatic fat content and improvements in NAFLD among obese patients. The degree of weight loss correlates with the reduction in liver fat. These findings underscore the clinical utility of bariatric surgery as a strategic intervention for managing NAFLD in obese individuals, potentially influencing clinical practice guidelines by integrating bariatric surgery as a viable treatment option for NAFLD-related hepatic conditions.

## 1. Introduction

Non-alcoholic fatty liver disease (NAFLD) has emerged as the most prevalent chronic liver condition worldwide, which is closely associated with escalating rates of obesity and metabolic syndrome [1,2]. Characterized by excessive fat accumulation in hepatocytes without significant alcohol consumption, NAFLD encompasses a spectrum of liver pathologies ranging from simple steatosis to non-alcoholic steatohepatitis (NASH), fibrosis, and cirrhosis [3,4]. The global burden of NAFLD is substantial, affecting approximately 25% of the adult population, and poses significant risks for liver-related morbidity and mortality [5,6]. As obesity rates continue to rise, so does the incidence of NAFLD, necessitating effective interventions to mitigate its progression [7].

Bariatric surgery has been recognized as the most effective treatment for morbid obesity, leading to substantial and sustained weight loss and improvement in obesity-related comorbidities [8]. Surgical procedures such as laparoscopic sleeve gastrectomy (LSG) and laparoscopic Roux-en-Y gastric bypass (LRYGB) have demonstrated significant metabolic benefits beyond weight reduction, including the amelioration of insulin resistance, dyslipidemia, and hypertension [9,10]. Importantly, bariatric surgery has been associated with histological improvements in NAFLD and NASH, suggesting a potential therapeutic role in reversing hepatic steatosis and preventing disease progression [11,12].

Magnetic resonance imaging proton density fat fraction (MRI-PDFF) has emerged as a non-invasive, accurate, and quantitative tool for assessing liver fat content [13]. Unlike traditional imaging modalities such as ultrasound or computed tomography, MRI-PDFF provides a reproducible and sensitive measure of hepatic steatosis, enabling the monitoring of changes in liver fat over time [14,15]. The utilization of MRI-PDFF in evaluating the effects of bariatric surgery on intrahepatic fat content offers a valuable opportunity to understand the temporal dynamics of NAFLD improvement post-intervention [16].

Despite the recognized benefits of bariatric surgery on NAFLD, the timing and extent of hepatic fat reduction post-surgery remain incompletely understood. Previous studies have reported significant decreases in liver fat content and volume following bariatric procedures, but the pattern of these changes and their correlation with weight loss and metabolic parameters require further elucidation [17,18]. Understanding the trajectory of hepatic fat reduction is crucial for optimizing patient management and monitoring therapeutic efficacy.

This systematic review aims to synthesize current evidence on the effects of bariatric surgery on intrahepatic fat changes as assessed by MRI-PDFF. By analyzing data from multiple studies, we seek to determine the extent and timing of hepatic fat reduction post-bariatric surgery, the differences between surgical procedures, and the correlation with weight loss and metabolic improvements. Additionally, this review will explore the utility of MRI-PDFF as a monitoring tool for NAFLD in the bariatric population. Given the increasing prevalence of NAFLD and the growing utilization of bariatric surgery, understanding the interplay between these conditions is of significant clinical importance. 

## 2. Materials and Methods

### 2.1. Eligibility Criteria

This systematic review included studies that evaluated the effects of bariatric surgery on intrahepatic fat content using MRI-PDFF. Inclusion criteria were as follows: (1) adult patients (≥18 years) with a diagnosis of NAFLD undergoing bariatric surgery; (2) assessment of liver fat changes using MRI-PDFF before and after surgery; (3) studies reporting quantitative data on liver fat fraction and relevant clinical parameters; and (4) randomized controlled trials, cohort studies, or case series with a minimum of ten participants. Exclusion criteria were as follows: (1) studies involving patients with significant alcohol intake (>20 g/day for women and >30 g/day for men), viral hepatitis, or other chronic liver diseases; (2) non-human studies; (3) studies without sufficient data on MRI-PDFF measurements; and (4) reviews, editorials, or conference abstracts without original data.

A comprehensive literature search was conducted across the PubMed, Scopus, and Web of Science databases from inception to October 2024. The search aimed to identify all relevant studies evaluating intrahepatic fat changes assessed by MRI-PDFF in patients undergoing bariatric surgery. Reference lists of included articles and relevant reviews were also screened to identify additional studies. In this study, grammar and spelling corrections were performed using an artificial intelligence language model ChatGPT (OpenAI, San Francisco, CA, USA).

### 2.2. Search Strategy

To optimize the breadth and depth of the literature search, a comprehensive and precise strategy was implemented using a blend of medical subject headings (MeSH) terms and specific keywords. The search focused on the intersection of bariatric surgery, MRI-PDFF techniques, and NAFLD. The key terms used included variations and synonyms to capture the broad spectrum of research in these areas. The search terms were: (“bariatric surgery” OR “gastric bypass” OR “sleeve gastrectomy” OR “metabolic surgery”) AND (“non-alcoholic fatty liver disease” OR “NAFLD” OR “hepatic steatosis” OR “liver fat accumulation”) AND (“MRI-PDFF” OR “magnetic resonance imaging proton density fat fraction” OR “liver fat fraction” OR “quantitative hepatic MRI”). Boolean operators were strategically employed to ensure a wide yet relevant array of articles, and truncations were used where appropriate to include all possible endings of a root word, thereby maximizing the search sensitivity and specificity.

### 2.3. Selection Process and Study Registration

Two independent reviewers screened the titles and abstracts of all retrieved articles for eligibility. Full-text articles were obtained for studies that met the inclusion criteria or when eligibility was unclear from the abstract. Discrepancies between reviewers were resolved through discussion or consultation with a third reviewer. The study protocol and selection process adhered to the Preferred Reporting Items for Systematic Reviews and Meta-Analyses (PRISMA) guidelines [19], ensuring transparency and reproducibility. The study was registered in the Open Science Framework with the registration code osf.io/uhnw8.

### 2.4. Data Collection Process

Data extraction was performed independently by two reviewers using a standardized data extraction form. Extracted data included study characteristics (author, year, country, study design), patient demographics (sample size, age, gender), type of bariatric surgery, baseline and follow-up MRI-PDFF measurements, liver volume, weight loss parameters, and changes in metabolic profiles. Authors were contacted for missing or unclear data when necessary. The extracted data were cross-checked for accuracy, and any discrepancies were resolved through consensus.

### 2.5. Risk of Bias and Quality Assessment

The quality of the included studies was assessed using the Newcastle–Ottawa Scale (NOS) for cohort studies. The NOS evaluates studies based on three domains: selection of participants, comparability of study groups, and ascertainment of outcomes. Each study was independently assessed by two reviewers, and discrepancies were resolved by consensus. NOS scores range from 0 to 9, with higher scores indicating better quality [20].

## 3. Results

### Study Selection and Study Characteristics

**Figure 1:**
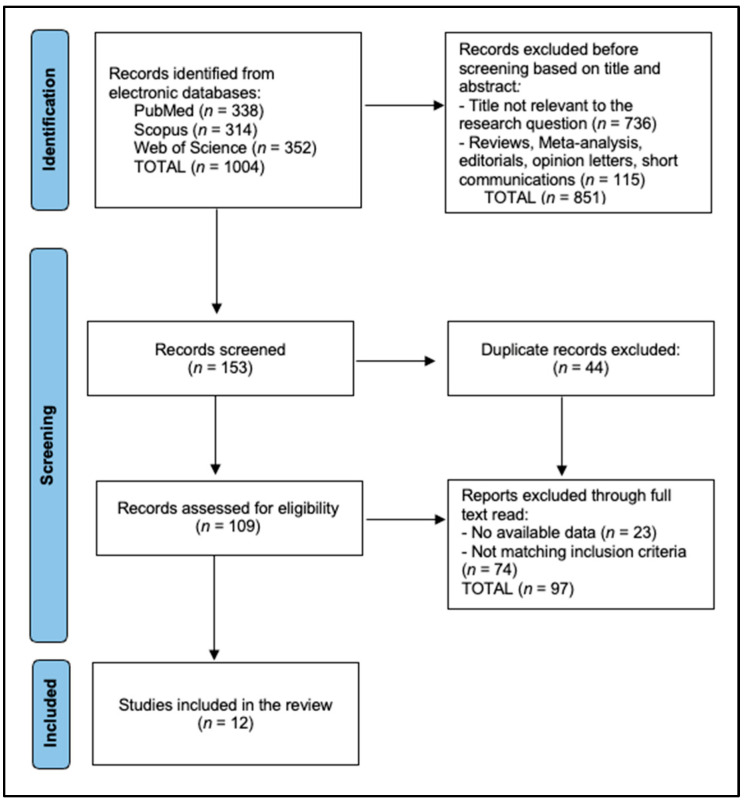
PRISMA flow diagram.

The current systematic review included a total of 12 prospective cohort studies conducted between 2013 and 2023 across five countries, a presented in Figure 1 [21,22,23,24,25,26,27,28,29,30,31,32]. The total sample size encompasses 613 participants, reflecting substantial research on the effects of bariatric surgery on intrahepatic fat content. The studies vary in sample size from 9 to 118 participants, offering both detailed individual assessments and broader population analyses. The types of bariatric surgery investigated include laparoscopic adjustable gastric banding, BioEnterics intragastric balloon, laparoscopic sleeve gastrectomy, and laparoscopic Roux-en-Y gastric bypass. Most studies have a follow-up duration of six months, providing sufficient time to observe significant physiological changes post-surgery. The Newcastle–Ottawa Scale (NOS) scores range from six to nine, indicating that the majority of studies are of moderate-to-high quality (Table 1). The highest score of nine was achieved by Fazeli Dehkordy et al. [29].

Table 2 shows that participants’ mean ages span from as young as 32 in Li et al. [27] to 54.3 in Syväri et al. [28], with a general trend towards middle-aged adults. Gender distribution varies, with a notable predominance of female participants in most studies, such as in Hedderich et al. [22] with a ratio of four males to fifteen females, and an even more skewed distribution in Fazeli Dehkordy et al. [29], where females outnumber males 102 to 16. This suggests a higher inclination or acceptance of bariatric surgery among females. Baseline BMIs are consistently high across all datasets, reflecting severe obesity levels, with Luo et al. [23] reporting the highest mean BMI at 45.3 ± 5.9 kg/m². Post-surgery, delta BMI values show notable reductions, indicating effective weight loss outcomes, such as −12.2 kg/m², as reported in Allen et al. [31], and −11.02 kg/m² reported in Bai et al. [30].

Table 3 illustrates the weight loss outcomes following bariatric surgery. All studies report significant weight loss, demonstrating the efficacy of bariatric procedures in achieving substantial reductions in body weight among severely obese patients. Weight loss percentages vary among the studies, from 18.2% to 24.6% of initial body weight. Excess weight loss percentages exceed 55% in several studies, indicating the proportion of weight lost beyond an individual’s ideal body weight. Delta weight indicates absolute weight loss, with reductions up to 34.6 kg.

The changes in intrahepatic fat content reported across various studies provide significant insights into the effectiveness of bariatric surgery in reducing liver fat, as quantified by MRI-PDFF (magnetic resonance imaging proton density fat fraction) values. Table 4 consolidates these changes, illustrating a broad spectrum of reductions in liver fat percentages post-surgery. For instance, Bai et al. [30] show one of the most substantial reductions, with a delta MRI-PDFF of −14%, moving from a baseline of 16.90 ± 9.45% to 2.91 ± 2.57%. Similarly, Pooler et al. [25] reported a decrease from 18.1 ± 8.6% to 4.9 ± 3.4%, yielding a −13.2% change. These reductions suggest that various forms of bariatric surgery, whether gastric bypass, sleeve gastrectomy, or other methods, are highly effective in decreasing hepatic steatosis, which is a critical factor in improving overall liver health and reducing the risks associated with non-alcoholic fatty liver disease (NAFLD).

The consistency in the decrease of MRI-PDFF values across most studies indicates a universal benefit of surgical interventions on liver fat content, regardless of patient demographics or the specific surgical technique used. For example, Luo et al. [23] reported a decrease from 16.6 ± 7.8% to 4.4 ± 3.4%, which is a delta of −12.2%. Meanwhile, Mamidipalli et al. [24] observed a change from 16.6 ± 7.2% to 5.6 ± 3.7%, translating to a −11% delta. These figures not only highlight the significant impact of bariatric surgery on reducing liver fat but also emphasize the potential for these procedures to aid in the reversal or improvement of conditions related to excessive hepatic fat, such as NAFLD and insulin resistance.

However, an exception noted in the data from Syväri et al. [28] indicates a unique outcome where a slight increase in MRI-PDFF values was observed, from 9.8 ± 9.7% to 10.2 ± 9.5%. This minor increase, marked by a 0.4% change, suggests that factors beyond the surgical procedure, potentially including lifestyle interventions or post-surgical dietary changes, may influence liver fat outcomes. This anomaly underscores the complexity of managing liver fat and the need for a holistic approach that includes both surgical and lifestyle modifications to achieve optimal results in hepatic health post-bariatric surgery (Table 4). 

## 4. Discussion

### 4.1. Summary of Evidence

The aggregation of data from these 12 studies provides compelling evidence that bariatric surgery leads to significant reductions in intrahepatic fat content in patients with NAFLD. The consistent decreases in MRI-PDFF values across diverse populations and surgical procedures underscore the effectiveness of bariatric surgery as a therapeutic intervention for hepatic steatosis associated with obesity. Significant weight loss achieved through bariatric procedures correlates strongly with reductions in liver fat content. Studies such as those by Luo et al. [23] and Allen et al. [31] report substantial delta BMI reductions and corresponding decreases in MRI-PDFF, highlighting the inter-relationship between weight loss and hepatic health.

MRI-PDFF serves as a valuable, non-invasive modality for quantifying liver fat content. Its consistent use and the significant reductions observed reinforce its utility in both clinical practice and research settings. Several studies reported high rates of resolution of steatosis and NASH. For instance, Bai et al. [30] noted a 95.2% cure rate in metabolic dysfunction-associated steatotic liver disease post-surgery. Allen et al. [31] observed that NASH resolved in all patients one year after bariatric surgery. These findings suggest that bariatric surgery not only reduces liver fat but may also reverse inflammatory processes associated with NASH.

In a similar manner to the study by Loy et al. [33], which examined the effects of the laparoscopic adjustable gastric band (LAGB) on NAFLD and metabolic syndrome (MS) in adolescents, the research conducted by Nixdorf et al. [34] focused on adult patients undergoing various forms of metabolic/bariatric surgery (MBS). Loy et al. [33] reported that, following LAGB, obese adolescents experienced a notable decrease in mean BMI from 48.8 kg/m^2^ pre-operatively to 36.8 kg/m^2^ at 24 months, alongside improvements in NAFLD scores from an average decrease of 0.68 at one year to 0.38 at two years, and a significant reduction in MS rates from 27% to 2% over the same period. Similarly, Nixdorf et al. [34] observed rapid improvements in metabolic dysfunction-associated steatotic liver disease (MASLD) and related steatohepatitis (MASH) within just three months post-surgery. They reported a median relative total weight loss of 20.1%, with a decrease in median BMI from 46.0 kg/m^2^ to 36.1 kg/m^2^, and significant reductions in liver stiffness measurements (LSM) and controlled attenuation parameter (CAP), which suggest improvements in liver fibrosis and steatosis. Furthermore, the reduction in liver injury markers such as ALT and gamma-glutamyl transferase underscores the swift therapeutic impact of MBS on liver health, paralleling the adolescent improvements seen with LAGB in the Loy et al. study [33].

In a comprehensive analysis of bariatric surgery’s impact on NAFLD, the study by Fakhry et al. [35] encompassed a large cohort, aggregating results from 21 studies with a total of 2374 patients. This systematic review and meta-analysis found substantial improvements in liver health post-surgery, notably an 88% improvement in steatosis, 59% in steatohepatitis, and a 30% improvement in liver fibrosis, showcasing the significant potential for bariatric procedures to alter the course of NAFLD. The Roux-en-Y gastric bypass was particularly effective, showing higher rates of improvement compared to other procedures. Similarly, the study by Kim et al. [36], although smaller in scale with 32 Korean patients, echoed these positive outcomes in a short-term follow-up of six months. Significant reductions were noted in controlled attenuation parameters (from 325.4 ± 55.9 dB/m to 267.1 ± 45.1 dB/m) and liver stiffness (from 7.4 ± 4.8 kPa to 5.3 ± 2.3 kPa), affirming the beneficial impact of bariatric surgery on liver steatosis and fibrosis. 

In a similar study, Ruiz-Tovar et al. [37] demonstrated significant improvements in metabolic-associated fatty liver disease (MAFLD) in morbidly obese women following RYGB combined with adherence to a Mediterranean-like diet. Magnetic resonance spectroscopy revealed a substantial reduction in the percentage of liver lipid content, from an average of 14.2% pre-operatively to 4.0% one year postoperatively (*p* < 0.001). Interestingly, those who adhered highly to the Mediterranean diet saw even greater improvements, highlighting the synergistic benefits of dietary management alongside surgical intervention. In a similar manner, the study by Kirkpatrick et al. [38] focused on a broader set of metabolic improvements following bariatric metabolic surgery in patients with non-alcoholic fatty liver disease. This retrospective analysis reported significant reductions in liver enzymes with a 57.6% decrease in ALT and a 47.7% decrease in AST one year after surgery. Moreover, there were notable improvements in metabolic parameters such as blood glucose, lipid levels, and HbA1c, emphasizing the multifaceted benefits of bariatric surgery beyond liver health alone.

The study by Głuszyńska et al. [39] explored the efficacy of laparoscopic sleeve gastrectomy in treating non-alcoholic fatty liver disease among morbidly obese patients over a one-year follow-up. It showed substantial improvements, with the percentage of liver lipid content dramatically reduced from a mean of 14.2% to 4.0%, and a significant decline in the non-alcoholic fatty liver fibrosis score from 0.2 to −1.6 (*p* < 0.0001). These results were paralleled by considerable weight loss metrics, emphasizing the surgery’s role in mitigating liver steatosis. Similarly, the BRAVES trial by Verrastro et al. [40] confirmed the superiority of bariatric-metabolic surgeries (Roux-en-Y gastric bypass and sleeve gastrectomy) over lifestyle interventions plus medical care in achieving histological resolution of NASH without worsening fibrosis. Notably, in an intention-to-treat analysis, resolution rates were significantly higher in the surgical groups (56% and 57%, respectively) compared to only 16% in the lifestyle modification group (*p* < 0.0001). Both studies underscore the potent impact of surgical interventions on liver health, offering substantial evidence that bariatric surgery is an effective strategy for NAFLD and NASH management, with durable benefits observed in liver function and structure.

The clinical utility of bariatric surgery in severely obese patients, as demonstrated across multiple studies, highlights significant outcomes in weight management and liver health improvement. These surgeries, including laparoscopic gastric bypass and sleeve gastrectomy, consistently yield substantial reductions in body mass index and weight, with some studies noting decreases up to 12.2 kg/m² in BMI and 34.6 kg in weight. Additionally, improvements in liver parameters, particularly liver fat content measured by MRI-PDFF, with reductions as high as 14%, underscore the dual benefits of these procedures. This evidence is invaluable in guiding healthcare providers to consider bariatric surgery not only for weight loss but also as a viable option for mitigating complications like non-alcoholic fatty liver disease, thereby supporting integrated treatment approaches for comprehensive patient care in clinical settings. 

### 4.2. Limitations

Despite the promising results, several limitations should be considered. The sample sizes of some studies are relatively small, which may limit the generalizability of the results. The heterogeneity in study designs, surgical procedures, and patient populations introduces variability that may affect the comparability of outcomes. The predominance of female participants may also limit the applicability of findings to male patients. Additionally, follow-up durations are generally limited to six months to one year, which may not capture the long-term sustainability of hepatic fat reduction.

## 5. Conclusions

Bariatric surgery is effective in significantly reducing intrahepatic fat content and improving liver health in obese patients with NAFLD. The substantial weight loss achieved through various bariatric procedures correlates with improvements in hepatic steatosis and overall metabolic health. MRI-PDFF serves as a reliable, non-invasive method for monitoring these changes. High-quality evidence supports bariatric surgery as a viable option in the management of NAFLD among obese individuals. Future studies with larger sample sizes and longer follow-up periods are needed to confirm these findings and to evaluate the long-term impact of bariatric surgery on liver disease progression.

## Figures and Tables

**Table 1:** Study characteristics and quality assessment.

Study No.	Author(s)	Country	Year	Sample Size (n)	Types of Bariatric Surgery	Follow-Up Duration	Quality Score (NOS)
1	Folini et al. [21]	Italy	2013	40	LAGB/BIB/Diet	6 months	7
2	Hedderich et al. [22]	Germany	2017	19	LSG/LRYGB	6 months	8
3	Luo et al. [23]	USA	2018	49	LSG/LRYGB/LAGB	6 months	8
4	Mamidipalli et al. [24]	USA	2020	54	LSG/LRYGB	6 months	8
5	Pooler et al. [25]	USA	2019	50	LSG/LRYGB/LAGB	6 months	8
6	Tan et al. [26]	Singapore	2023	9	LSG	6 months	6
7	Li et al. [27]	China	2020	69	LSG/LRYGB	3 months	7
8	Syväri et al. [28]	Germany	2021	32	Lifestyle Intervention	1 year	7
9	Fazeli Dehkordy et al. [29]	USA	2018	118	VLCD + WLS	Up to 6 months	9
10	Bai et al. [30]	China	2023	44	LSG	6 months	7
11	Allen et al. [31]	USA	2019	38	Bariatric Surgery	1 year	8
12	Sun et al. [32]	China	2021	91	LSG	~100 days	7

LAGB—laparoscopic adjustable gastric banding; BIB—BioEnterics intragastric balloon; LSG—laparoscopic sleeve gastrectomy; LRYGB—laparoscopic Roux-en-Y gastric bypass; VLCD—very low-calorie diet; WLS—weight loss surgery.

**Table 2:** Patient demographics and baseline characteristics.

Study	Mean Age (Years)	Gender (M/F)	Baseline BMI (kg/m²)	Delta BMI (kg/m²)
Folini et al. [21]	43.6 ± 12.2	2/16	43.8 ± 6.62	−5.6
Hedderich et al. [22]	41.4 ± 12.5	4/15	44.1 ± 5.2	−10.3
Luo et al. [23]	50.9 ± 10.8	7/42	45.3 ± 5.9	−10.9
Mamidipalli et al. [24]	52 ± 12	10/44	42.3 ± 5.0	−8
Pooler et al. [25]	51.0 ± 11.2	7/43	44.9 ± 6.5	−10.4
Tan et al. [26]	45.1 ± 9.0	5/4	39.7 ± 5.3	−7.3
Li et al. [27]	32 ± 8.67	14/55	37.92 ± 6.58	−8.05
Syväri et al. [28]	54.3	14/18	Not specified	Variable
Fazeli Dehkordy et al. [29]	48.0 ± 13.0	16/102	43.4 ± 6.2	Not specified
Bai et al. [30]	Not specified	Not specified	38.91 ± 5.29	−11.02
Allen et al. [31]	50 (Median)	5/33	44.6	−12.2
Sun et al. [32]	Not specified	18/73	38.4	−8.1

**Table 3:** Weight loss outcomes.

Study	Weight Loss (%)	Excess Weight Loss (%)	Delta Weight (kg)
Folini et al. [21]	Significant (*p* = 0.001)	Not reported	−14.1
Hedderich et al. [22]	24.5 ± 6.3%	58.7 ± 19.7%	−32.5
Luo et al. [23]	Significant (*p* < 0.001)	55.6 ± 19.0%	−31.7
Mamidipalli et al. [24]	Not reported	Not reported	−24.3
Pooler et al. [25]	24.6% weight loss	Not reported	−34.6
Tan et al. [26]	18.2% weight loss	Not reported	−19.4
Li et al. [27]	21.3% weight loss	Not reported	−22.7
Syväri et al. [28]	Variable weight change	Not applicable	−1.2 to +1.4
Fazeli Dehkordy et al. [29]	Not specified	Not specified	Not specified
Bai et al. [30]	Significant decrease	Not specified	−26.67
Allen et al. [31]	Not specified	Not specified	Not specified
Sun et al. [32]	Median weight loss of 23.1 kg	EWL: 58.7%	−23.1

**Table 4:** Changes in intrahepatic fat content.

Study	Baseline MRI-PDFF (%)	Follow-Up MRI-PDFF (%)	Delta MRI-PDFF (%)
Folini et al. [21]	16.7 ± 10.91	7.6 ± 9.76	−9.1
Hedderich et al. [22]	10.1 ± 9.7	3.2 ± 2.2	−6.9
Luo et al. [23]	16.6 ± 7.8	4.4 ± 3.4	−12.2
Mamidipalli et al. [24]	16.6 ± 7.2	5.6 ± 3.7	−11
Pooler et al. [25]	18.1 ± 8.6	4.9 ± 3.4	−13.2
Tan et al. [26]	14.1 ± 7.4	4.9 ± 2.2	−9.2
Li et al. [27]	14.64 ± 9.02	5.74 ± 5.04	−8.9
Syväri et al. [28]	9.8 ± 9.7	10.2 ± 9.5	0.4
Fazeli Dehkordy et al. [29]	13.6% (right lobe)	4.20%	−9.4
Bai et al. [30]	16.90 ± 9.45	2.91 ± 2.57	−14
Allen et al. [31]	10.0 (median)	2.7 (median)	−7.3
Sun et al. [32]	13.60 (median)	3.44 (median)	−10.16

MRI—magnetic resonance imaging; PDFF—proton density fat fraction.

## Data Availability

Data sharing is not applicable. No new data were created or analyzed in this study.
